# Methicillin-resistant *Staphylococcus aureus* (MRSA) infection in hospitalized patients is dominated by community-acquired strains: genomic epidemiological evidence

**DOI:** 10.1371/journal.pone.0354017

**Published:** 2026-07-30

**Authors:** Jingxia Dang, Yuhui Geng, Ting Pan, Ping Zhang, Mingbo Chen, Dongfeng Pan, Peifeng Liang

**Affiliations:** 1 School of Public Health, Ningxia Medical University, Yinchuan, China; 2 Ningxia Key Laboratory of Environmental Factors and Chronic Disease Control, Yinchuan, China; 3 Hospital-Acquired Infection Control Department, General Hospital of Ningxia Medical University, Yinchuan, China; 4 Department of Emergency Medicine, People’s Hospital of Ningxia Hui Autonomous Region, Ningxia Medical University, Yinchuan, China; 5 Department of Medical Affairs, People’s Hospital of Ningxia Hui Autonomous Region, Ningxia Medical University, Yinchuan, China; USP FMVZ: Universidade de Sao Paulo Faculdade de Medicina Veterinaria e Zootecnia, BRAZIL

## Abstract

**Objective:**

This study aimed to systematically investigate the molecular epidemiological characteristics of methicillin-resistant *Staphylococcus aureus* (MRSA) in Ningxia hospitals, to elucidate their genetic evolutionary relationships, and to delineate the genomic and phenotypic profiles of the dominant lineages.

**Methods:**

Clinical isolates of MRSA strains collected between **01/01/2024 and 30/06/2024** were analyzed, employing second-generation gene sequencing technology, combined with MLST and SCC*mec* typing, along with evaluation of drug resistance and virulence genes. A phylogenetic tree was constructed to analyze strain homology.

**Results:**

A total of 74 non-duplicate *Staphylococcus aureus* strains (67 MRSA and 7 MSSA) were collected. The most common clonal strain was ST59-IVa, accounting for 46.27%. This strain exhibited a high prevalence of resistance genes *mecA* and *blaZ*, at 91.04%. All five ST22-IVa strains were found to lack *mecA* and *erm* genes but showed β-lactam resistance, while possessing both *lukS/F-PV (*PVL*)* and *tsst-1* virulence genes, indicating a significant toxicity risk. Genetic evolution analysis revealed that ST3355, ST4513, and ST59 were closely related, all belonging to SCC*mec* types IVa; the other ST types exhibited mutations at various loci, with ST5 as the central node, resulting in a wider array of ST and SCC*mec* typing.

**Conclusion:**

The ST59-IVa clone is the predominant MRSA strain in Ningxia hospitals, exhibiting multidrug resistance and virulence gene profiles consistent with national trends. However, the emergence of hypervirulent ST22-IVa strains with atypical resistance mechanisms warrants increased vigilance. We recommend enhancing the rational use of antibiotics in hospitals and implementing molecular surveillance for these highly virulent strains.

## 1 Introduction

MRSA represents a significant pathogen characterized by multidrug resistance and high pathogenicity [1]. Since its initial identification, the prevalence of MRSA has shown a consistent increase. Annual detection rates in clinical *Staphylococcus aureus* isolates have been 28.4% [2]. The dissemination of MRSA has transitioned from hospital settings to community environments, leading to the emergence of two primary epidemiological categories: hospital-acquired MRSA (HA-MRSA) and community-acquired MRSA (CA-MRSA) [3,4]. In recent years, the boundary between HA-MRSA and CA-MRSA has become increasingly blurred, the prevalence of CA-MRSA has increased significantly, and has become widespread into hospitals to become a major pathogen in the hospital environment because it carries smaller mobile genetic elements and virulence factors, as well as exhibits greater transmissibility and pathogenicity [5,6].

Studies have indicated that the clonal distribution of MRSA exhibits considerable geographic variability. For instance, ST80‑IV predominates in Europe, ST1‑IV/ST8‑IV in North America, and ST59‑IV/V in China and several other Asian nations [7,8]. Recent years have also seen dynamic shifts in the prevalent MRSA clones within China, with ST59-IV replacing ST239-III as the dominant lineage, accounting for over 40% of isolates [9]. Notably, regional disparities persist: ST5-II dominates in Hainan [10], whereas ST59 constitutes 55.4% of isolates in Fujian [11]. Additionally, highly virulent ST22 clones accounted for a rising proportion of infections in children in the east [12], suggesting the need to be alert to the risk of its cross-regional transmission. The molecular characteristics of MRSA in Ningxia have not been systematically investigated, which may be influenced by factors such as healthcare resources and antibiotic usage patterns, necessitating clarification of its unique epidemiological features [13].

Moreover, MRSA exhibits resistance to the majority of antibiotics typically employed for the treatment and management of its infections, primarily due to the *mecA* gene it harbors, which encodes the low-affinity penicillin-binding protein PBP2a, thereby diminishing its affinity for β-lactam antibiotics [14,15]. Beyond its intrinsic drug resistance, MRSA infection is associated with substantial clinical and economic burdens, including prolonged hospitalization, higher medical costs, and increased mortality [16]. The proliferation of various staphylococcal clones carrying multiple virulence and antibiotic resistance factors has emerged as a public health concern [17–19]. Previous studies have indicated that MRSA isolates typically harbor multiple virulence genes associated with diverse functions. For example, the gene encoding for the leukocidin Panton-Valentine (PVL) is more prevalent in ST338, CC30, CC398, ST8, and CC22, while the toxic shock syndrome toxin-1 (*tsst-1*) is linked to ST5 [9]. Given the evolving epidemiology of MRSA, the emergence of new clonal strains in different geographical regions underscores the necessity for ongoing surveillance of MRSA resistance in the environment, molecular characterization of strains, and monitoring of genetic evolution.

This research employed whole-genome sequencing (WGS) to investigate the clonal distribution, drug resistance genes, virulence factors, and other attributes of MRSA within the hospital environment of Ningxia. Unlike previous local studies that relied on fragmentary typing, WGS enables precise identification of novel sequence types, genome-wide detection of resistance and virulence determinants, and accurate reconstruction of genetic evolution at single-nucleotide resolution. The objective was to achieve a thorough understanding of the prevalence of MRSA in healthcare settings, thereby facilitating effective management of MRSA transmission and infection, as well as informing the judicious use of antimicrobial agents. Furthermore, the findings aim to provide a scientific foundation for the formulation of rational infection prevention and control strategies.

## 2 Materials and methods

### 2.1 strain collection and identification

Clinical specimens were accessed for research purposes from symptomatic patients at three tertiary hospitals in Yinchuan, China (General Hospital of Ningxia People’s Hospital, Xixia Branch Hospital, and the Emergency Medical Center) between **01/01/2024 and 30/06/2024**. Sampling covered key clinical departments-Intensive Care Unit (ICU), Respiratory Medicine, Emergency Medicine, and General Surgery-to ensure broad clinical and geographical representation.

Initial bacterial identification was performed using matrix-assisted laser desorption/ionization time-of-flight mass spectrometry (MALDI-TOF MS; VITEK® MS, bioMérieux, France). All confirmed *Staphylococcus aureus* isolates were subsequently screened for methicillin resistance using cefoxitin disk diffusion according to Clinical and Laboratory Standards Institute (CLSI) guidelines [20,21] and by PCR detection [22] of the *mecA* gene. A total of 74 non-duplicate *S. aureus* clinical isolates (from 74 different patients) were included in the final analysis, comprising 67 MRSA and 7 MSSA strains. The inclusion and exclusion criteria for patient selection are detailed in S1 Table in S1 File. All strains were preserved at −80°C for subsequent analysis.

### 2.2 Classification of CA-MRSA and HA-MRSA

The following inclusion criteria for CA-MRSA were employed [23]: (i) MRSA identified in an outpatient setting or within 48 hours of hospital admission; (ii) no history of hospitalization, surgery, dialysis, or residence in a long-term care facility in the 12 months preceding the culture; and (iii) no indwelling catheter or percutaneous medical device at the time of culture. HA-MRSA was defined as any isolate that did not meet the CA-MRSA criteria, including isolates obtained >48 hours after admission or from patients with documented healthcare-associated risk factors.

### 2.3 Antimicrobial susceptibility testing (AST)

Antimicrobial susceptibility testing was performed for all 74 *S. aureus* isolates using the broth microdilution method, strictly following CLSI M07 guidelines. The antibiotic panel was selected according to the CLSI M100 guidelines for *Staphylococcus aureus***.** The minimum inhibitory concentrations (MICs) of the following antibiotics were determined: cefoxitin (4 µg/mL as the breakpoint for MRSA classification), oxacillin, penicillin, erythromycin, clindamycin, and levofloxacin. *Staphylococcus aureus* ATCC 29213 was used as the quality control strain.

### 2.4 Whole genome sequencing analysis

#### 2.4.1 DNA extraction and quality control.

Genomic DNA was extracted from bacterial cultures using the cetyltrimethylammonium bromide (CTAB) method. DNA quantity and quality were assessed using a Quant-iT PicoGreen dsDNA Assay Kit (Thermo Fisher Scientific, USA) and a NanoDrop spectrophotometer (Thermo Fisher Scientific, USA) with acceptance criteria of A260/280 = 1.8–2.0 and A260/230 ≥ 2.0; Sequencing libraries were constructed from 100 ng of genomic DNA (concentration ≥ 50 ng/μL) using the Illumina TruSeq Nano DNA LT Library Prep Kit (Illumina, USA) following the manufacturer’s protocol.

#### 2.4.2 Library preparation, sequencing and genome assembly.

Libraries were prepared using the Illumina TruSeq Nano DNA LT Library Prep Kit (Illumina, USA). Genomic DNA was fragmented with a Covaris ultrasonicator (Covaris, USA), followed by end repair, adapter ligation, and PCR enrichment. Library quality and concentration were assessed using an Agilent Bioanalyzer 2100 (Agilent, USA), and qualified libraries were diluted to 4–5 pM before cluster generation. Paired-end 150‑bp sequencing was carried out on the Illumina NovaSeq 6000 platform (Illumina, USA). Raw reads were filtered by fastp (v0.20.0) to remove adapters and low-quality reads with a Phred quality score < 20. De novo genome assembly was performed with SPAdes (v3.15.5) under default parameters. Scaffolds longer than 500 bp and with an average sequencing depth over 10 × were kept for subsequent analysis. The assembled sequences were polished using Pilon (v1.24). Species identification was implemented via BLASTn against the NCBI NT database with an E-value cutoff of 1e-5, and the top 10 homologous sequences per sample were retained.

### 2.5 Bioinformatic analysis

Multi locus sequence typing (MLST) was conducted utilizing the PubMLST database [24]; while the determination of SCC*mec* types was performed through the SCC*mec*Finder tool [25]. Virulence factors were identified by BLAST against the Virulence Factors of Pathogenic Bacteria (VFDB) database [26] using thresholds of ≥80% identity and ≥60% coverage. Resistance genes were predicted using the ResFinder database [27] with default thresholds (≥90% nucleotide identity, ≥ 60% coverage), and results were cross-checked with the Comprehensive Antibiotic Resistance Database [28] (CARD).

### 2.6 SNP detection and evolutionary analysis

Single nucleotide polymorphism (SNP) detection was performed following the GATK v4.2 best-practice workflow. Reads were realigned around insertions and deletions (InDels) using RealignerTargetCreator and IndelRealigner to ensure variant calling accuracy. High-confidence SNPs were identified using UnifiedGenotyper with parameters set to stand_call_conf = 30 and stand_emit_conf = 10, followed by functional annotation using ANNOVAR. SNPs were filtered using GATK’s recommended hard‑filtering criteria (QD < 2.0, FS > 60.0, MQ < 40.0). A maximum-likelihood phylogenetic tree was constructed from the core genome SNP matrix via FastTree and visualized with MEGA11. Branch support was assessed using the bootstrap method (1,000 replicates; nodes with ≥70% support were considered reliable). A minimum spanning tree was generated using PHYLOViZ based on the eBURST algorithm, and genetic relationships among isolates were further interpreted in combination with MLST typing results.

### 2.7 Ethics approval and consent to participate

This study was approved by the Ethics Committee of the People’s Hospital of Ningxia Hui Autonomous Region (Approval No: Ethics [2023]-GZR-016). Clinical patients have signed informed consent. This study was performed in accordance with the ethical standards as laid down in the 1964 Declaration of Helsinki and its later amendments.

## 3 Results

### 3.1 Clinical characteristics of patients

**Fig 1:**
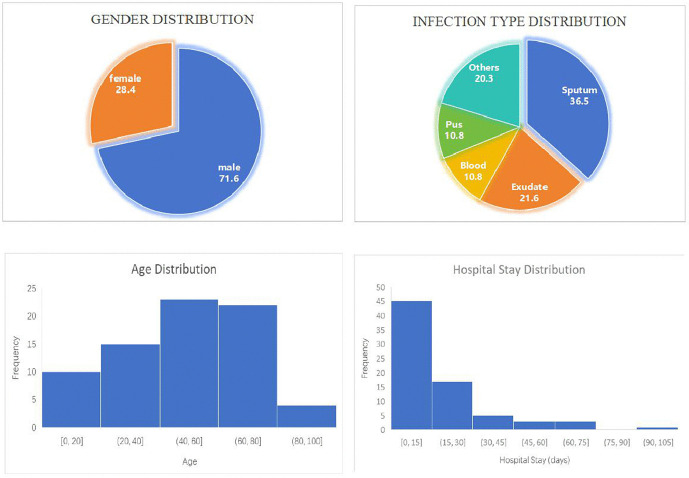
Clinical characteristics of patients with *S. aureus* infections (n = 74).

The median age of the patients from whom the 74 strains analyzed in this study were derived was 53 years, the gender distribution (male/female) was 53/21 (71.6%/28.4%). The most prevalent type of infection was sputum, accounting for 36.5% of cases, followed by secretions at 21.6%. Additionally, the median duration of hospitalization for these patients was 14 days (**Fig 1**). 42 of the 67 MRSA isolates (62.7%) were classified as CA‑MRSA, and the remaining 25 isolates (37.3%) were classified as HA‑MRSA. A detailed breakdown for each isolate is presented in S2 Table in S1 File.

### 3.2 MLST typing

**Fig 2:**
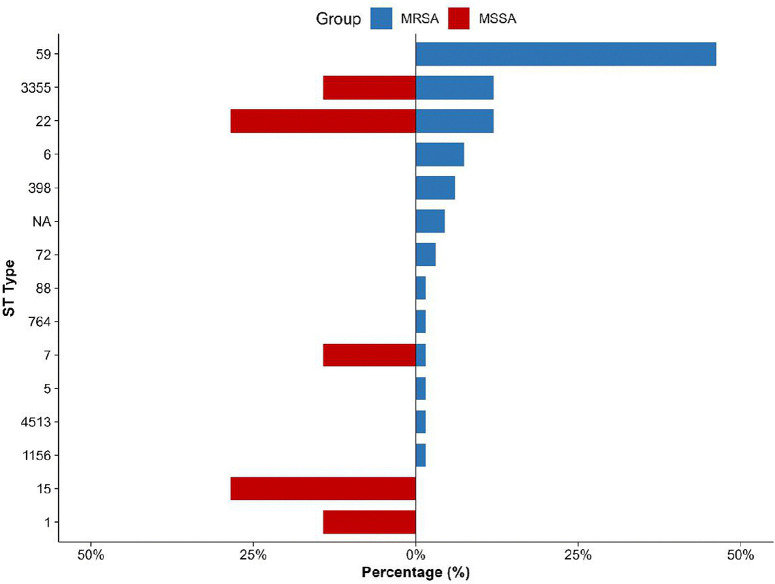
MLST typing distribution of 74 *S. aureus* isolates. ST59 (46.27%) was the dominant type. Percentages are calculated based on the total number of MRSA isolates (n = 67). Percentages for MSSA isolates are calculated based on the total number of MSSA isolates (n = 7). NA: New sequence type.

Molecular characterization of the 74 clinical isolates confirmed that 67 (90.54%) exhibited phenotypic methicillin resistance (based on cefoxitin testing) and were thus classified as MRSA, while 7 (9.46%) were MSSA. MLST-based analysis revealed that the MRSA strains comprised 12 established sequence types (ST) and along with 2 novel ST types (named NA), with ST59 being the dominant type (31/67, 46.27%), followed by ST22 and ST3355 (both 8/67, 11.94%). The MSSA strains encompassed 5 recognized ST types: ST22 (2 strains), ST15 (2 strains), ST3355 (1 strain), ST7 (1 strain), and ST1 (1 strain). In total, ten ST22 strains were identified (8 MRSA and 2 MSSA). (**Fig 2**).

### 3.3 SCC*mec* typing

By analyzing the SCC*mec* typing of 67 MRSA strains, six major typing types were identified, of which one strain was unclassified and defined as SCC*mec* NT 1.49% (1/67). The SCC*mec* types IVa type was the predominant typing type 77.61% (52/67), followed by the SCC*mec* types V 8.96% (6/67) the SCC*mec* types IVc 5.97% (4/67), SCC*mec* types II 2.99% (2/67), and one strain each of SCC*mec* types Vc and XII (1.49% each), (**Table 1**).

**Table 1:** Molecular typing of 67 MRSA strains.

ST	SCC*mec* type	strain number (n)	component ratio (%)
59	IVa	31	46.27
3355	IVa	8	11.94
22	IVa	5	7.46
	V	2	2.99
	IVc	1	1.49
6	IVa	5	7.46
398	V	2	2.99
	Vc	1	1.49
	XII	1	1.49
72	IVc	2	2.99
5	V	1	1.49
7	V	1	1.49
88	IVc	1	1.49
764	II	1	1.49
4513	IVa	1	1.49
1156	NT^*^	1	1.49
NA	IVa	2	2.99
	II	1	1.49
overall		67	100.00

Percentages are calculated based on the total number of MRSA isolates (n = 67). * Represents unclassified SCC*mec* type.

The integration of results from MLST and SCC*mec* typing revealed that the predominant clonal strain among the 67 MRSA isolates was ST59-IVa 46.27% (31/67). This was followed by ST3355-IVa 11.94% (8/67), ST22-IVa and ST6-Iva each accounted for 7.46% (5/67). The remaining ST22 MRSA strains were typed as V (2 strains) and IVc (1 strain), (**Table 1**).

### 3.4 Identification of drug resistance genes

In this investigation, a total of 14 drug resistance genes in 7 classes were detected in 67 MRSA strains, among which the methicillin resistance gene *mecA* and penicillin resistance gene *blaZ* had the highest detection rate of 91.04%, followed by the aminoglycoside resistance gene *ant(6)-Ia* (46.27%), as well as the aminoglycoside resistance genes *aph(3’)-III* and macrolide- Lincosamide-streptomycin resistance genes *erm(B)* were both detected at 44.78%, the *erm(C)* gene was identified in 10.45%, while the remaining resistance genes were detected at rates of less than 10% (**Table 2**).Notably, among the 67 phenotypically MRSA isolates, six were confirmed to lack the *mecA* gene by both PCR and whole‑genome sequencing: five ST22-IVa isolates and one ST398-XII isolate. No other known *mec* homologues (*mecB*, *mecC*, *mecD*) were detected in these six isolates. All six exhibited phenotypic resistance to cefoxitin and oxacillin, suggesting the presence of alternative, *mecA*‑independent resistance mechanisms. They were classified as MRSA solely based on their phenotypic cefoxitin resistance following CLSI guidelines, independent of *mecA* carriage.

**Table 2:** Carriage of drug resistance genes in 74 strains of *Staphylococcus aureus.*

Class of drugs	drug-resistance gene	MRSA n= 67 (%)	MSSA n = 7 (%)
Methicillin resistance	*mecA*	61 (91.04)	0 (0.00)
penicillin resistance	*blaZ*	61 (91.04)	6 (85.71)
aminoglycoside resistance	*ant(6)-Ia*	31 (46.27)	0 (0.00)
	*aph(3’)-III*	30 (44.78)	0 (0.00)
	*aac(6’)-aph(2”)*	6 (8.96)	1 (14.29)
	*aadD*	3 (4.48)	1 (14.29)
	*ant(9)-Ia*	2 (2.99)	0 (0.00)
Macrolide-lincosamide-streptomycin resistance	*erm(B)*	30 (44.78)	0 (0.00)
	*erm(C)*	7 (10.45)	2 (28.57)
	*erm(A)*	2 (2.99)	0 (0.00)
Tetracycline resistance	*tet(K)*	3 (4.48)	1 (14.29)
	*tet(M)*	2 (2.99)	0 (0.00)
lincosamide resistance	*lnu(A)*	3 (4.48)	1 (14.29)
Bleomycin resistance	*bleO*	1 (1.49)	0 (0.00)
Fusidic acid resistance	*fusB*	0 (0.00)	1 (14.29)

Among the seven MSSA strains, a total of 7 resistance genes in 6 classes were identified. Notably, the penicillin resistance gene *blaZ* exhibited the highest detection rate at 85.71%. This was followed by the macrolide-lincosamide-streptomycin resistance gene *erm(C)* which had a detection rate of 28.57%. The remaining genes, including *aac(6’)-aph(2”)*, *aadD*, *tet(K)*, *lnu(A)*, and *fusB*, were each detected at a rate of 14.29%, (**Table 2**).

### 3.5 Characterization of virulence genes of ST59, ST3355 and ST22 typing

**Fig 3:**
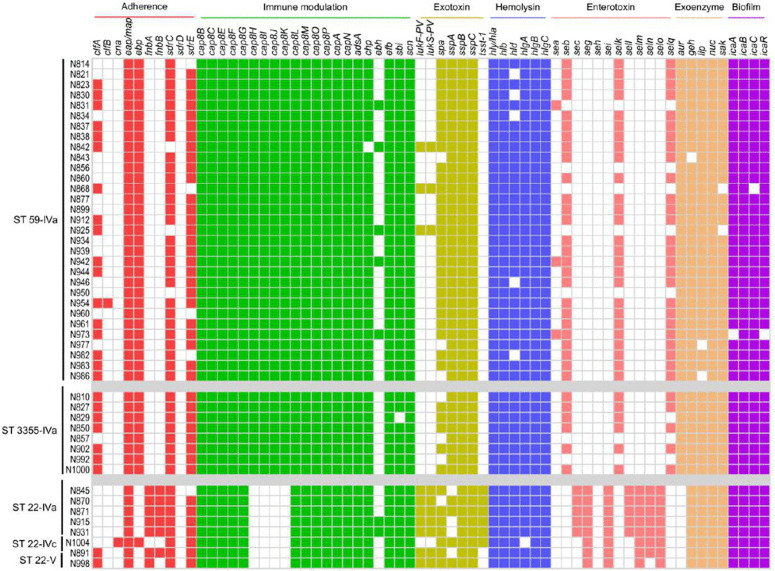
Distribution of virulence genes in ST59, ST3355, and ST22 typed MRSA strains. **Abbreviations: PVL:** Panton-Valentine leucocidin, ***tsst-1***: Toxic shock syndrome toxin-1, ***hla*/hly**: α-hemolysin, ***hlb***: β-hemolysin, ***hlg***: γ-hemolysin, ***cap8***: Capsular polysaccharide synthesis gene cluster, ***seb*/*selk*/*selq***: Staphylococcal enterotoxins B/K/Q, ***sec*/*seg*/*sei*/*selm*/*seln*/*selo***: Enterotoxin subtypes C/G/I/M/N/O.

An examination of the virulence factors present in MRSA strains revealed distinct characteristics among the three sequence types (STs): ST59, ST3355, and ST22. All isolates of ST59 were found to possess hemolysin-associated genes (*hly/hla, hlb, hlg*), in addition to the *cap8* -type virulence factor linked to capsular polysaccharide synthesis. Notably, three strains (9.68%) of ST59 also contained the *lukS/F-PV* (Panton-Valentine leukocidin, PVL) gene. The predominant enterotoxin profile identified among all MRSA isolates was *seb-selk-selq*, present in 35.82% of the isolates (24/67). Importantly, none of the ST59 strains exhibited the toxic shock syndrome toxin-1 (*tsst-1*) gene. (**Fig 3**).

In comparison, strain ST3355 exhibited a virulence gene profile similar to that of ST59; however, neither the PVL nor the *tsst-1* genes were detected, and the prevalence of the *seb*-*selk*-*selq* enterotoxin profile was significantly lower at 6.76%. Among the eight MRSA-ST22 strains, the *lukS/F-PV* gene was identified in seven (87.5%). Notably, all five ST22-IVa strains contained both the *lukS/F-PV* and *tsst-1* genes, indicating a hypervirulent profile. Importantly, these five strains are also the *mecA*‑negative isolates, demonstrating a unique combination of high virulence and atypical β‑lactam resistance independent of known *mec* genes. The enterotoxin gene profile *sec-seg-sei-selm-seln-selo* was predominant in ST22 strains. Furthermore, all isolates exhibited varying degrees of additional virulence factors, including adhesion factors, immunomodulatory factors, exotoxins, exoenzymes, and factors associated with biofilm formation. (**Fig 3**).

### 3.6 Drug resistance genes and phenotypes of ST59, ST3355 and ST22 typing

**Fig 4:**
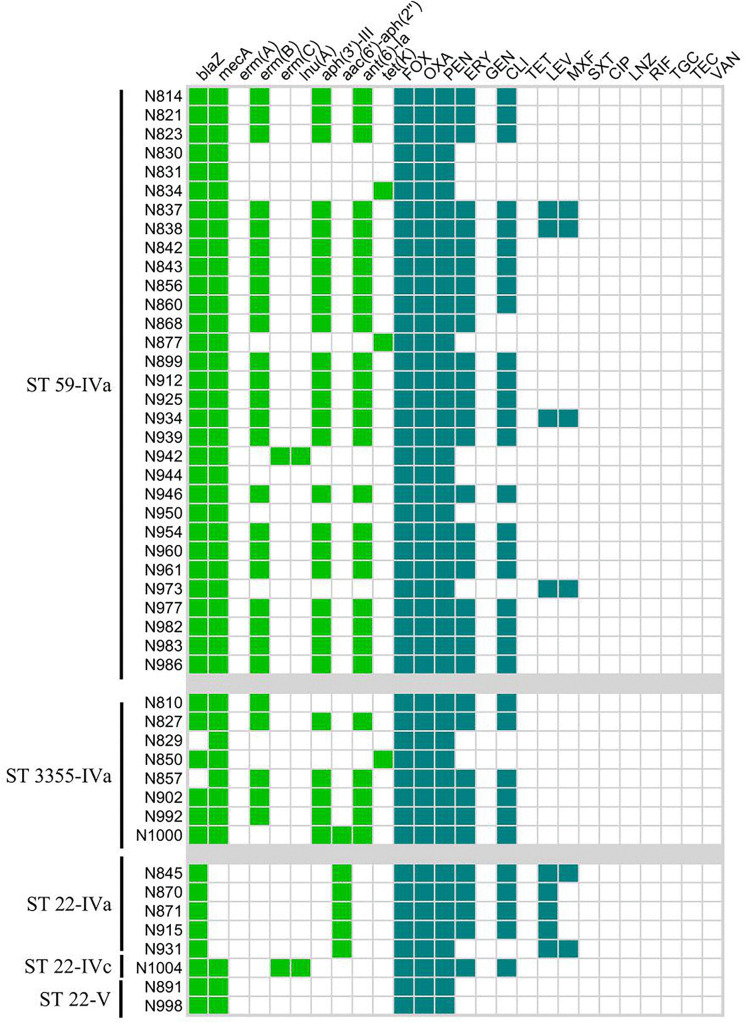
Resistance genes and resistance phenotypes of ST 59, ST 3355, and ST 22 typed MRSA strains. **Abbreviations: FOX:** Cefoxitin (β-lactam), **OXA**: Oxacillin (β-lactam), **PEN**: Penicillin, **ERY**: Erythromycin (macrolide), **CLI**: Clindamycin (lincosamide), **LVX**: Levofloxacin (fluoroquinolone). **Resistance gene annotation:** *blaZ*: penicillins (PEN); *mecA*: β-lactams (FOX, OXA); *erm(B/C)*: macrolides and lincosamides (ERY, CLI); *aac(6’)-aph(2”)*: aminoglycosides (not tested in this panel).

Drug susceptibility testing revealed that strain ST59 demonstrated resistance to cefoxitin, benzathine and penicillin. All strains encoding for the *erm(B)* gene exhibited resistance to erythromycin and clindamycin, Separately, some of these strains also displayed resistance to levofloxacin and moxifloxacin. The resistance genes and phenotypic resistance characteristics of strain ST3355 were found to be analogous to those of strain ST59. Although aminoglycoside resistance genes, specifically *aph(3’)-III* and *ant(6)-Ia*, were detected in both ST59 and ST3355, neither strain exhibited the corresponding resistance phenotypes. The five ST22‑IVa strains lacked *mecA* and possessed only two resistance genes, *blaZ* and *aac(6’)-aph(2”)*, yet still demonstrated resistance to cefoxitin, oxacillin, penicillin, erythromycin, and clindamycin, with all ST22-IVa strains being resistant to levofloxacin. Additionally, ST22-V, which contains only the *blaZ* and *mecA* genes, exhibited resistance phenotypes to cefoxitin, benzoxiline, and penicillin. (**Fig 4**)**.**

### 3.7 Genetic evolution analysis

**Fig 5:**
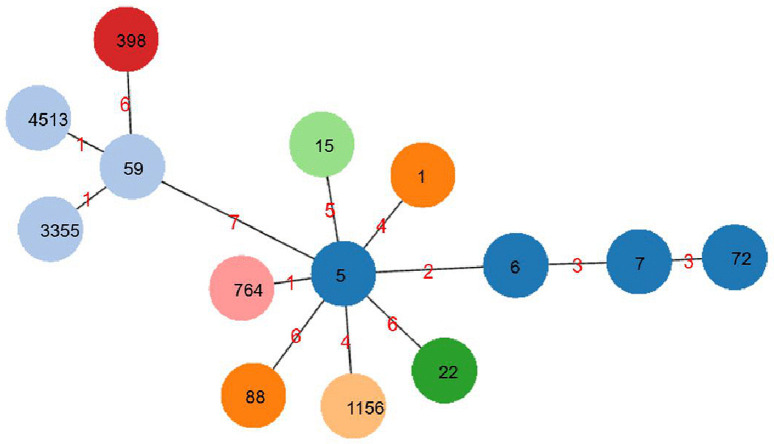
Minimum spanning tree for 74 strains of *Staphylococcus aureus*. represents the affinities of the 74 *S. aureus* strains identified by MLST typing. All 14 STs are represented by a colored circle. STs connected by a line belong to the same cluster, and the number on the connecting line represents the variant locus.

Based on MLST typing, a minimum spanning tree was constructed for 74 strains of *Staphylococcus aureus*, the kinship diagram revealed that among the 14 ST classifications, ST5 and ST59 served as the ancestral ST types. The remaining ST types exhibited mutations at various loci in comparison to these ancestral strains. Notably, distinct mutations in housekeeping genes were observed originating from ST59, with ST3355 and ST4513 exhibiting single-site mutations, while ST398 displayed multiple-site mutations. The ST5 ancestral strain demonstrated a greater diversity of housekeeping gene mutations, resulting in the emergence of eight ST sub-types. Among these, ST764 was characterized by a single-site mutation, ST6 by a double-site mutation, and the other ST types exhibited multiple-site mutations. (**Fig 5**)**.**

**Fig 6:**
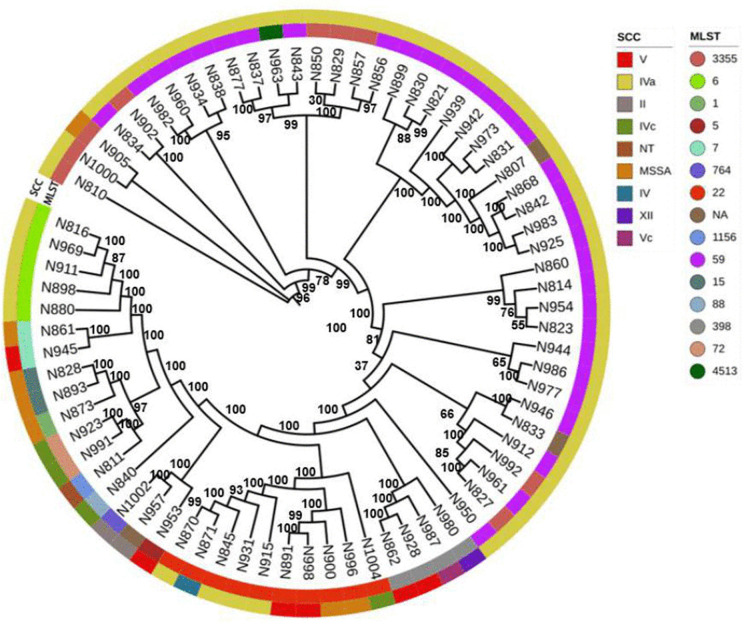
Phylogenetic tree of 74 *Staphylococcus aureus* strains based on core-genome single nucleotide polymorphisms (SNPs), covering 88.7% of the core genome with a total of 15,328 SNPs. Branch colors represent SCCmec and MLST typing (NA: new sequence type; NT: SCCmec type not defined). The scale bar is provided in S1 Fig in S1 File.

The phylogenetic tree constructed based on core SNP values reveals that all 74 *S. aureus* strains are situated within the same evolutionary branch, suggesting possible close genetic relatedness among the Ningxia isolates. ST3355 and ST4513 were closely related to ST59, as were the two untyped strains (NA in Table 1, internal IDs N807 and N833), all sharing the SCC*mec* types IVa. In contrast, ST398 was distinctly separated from the ST59 branch and exhibited diverse SCC*mec* types (V, Vc, XII). The genetic variation within the ST22 cluster was relatively stable, with no other ST types within this branch. Other samples closely related to ST5 showed variations at different loci and exhibited greater diversity in both ST and SCC*mec* types. (**Fig 6**)**.**

## 4 Discussion

### 4.1 Epidemiological characterization of dominant clonotypes

In this study, 42(62.7%) of the 67 MRSA strains were classified as CA-MRSA based on epidemiologic and genomic criteria, indicating the rapid dissemination of CA-MRSA within hospitals and underscoring its growing public health threat. The study identified ST59 (31/74, 41.89%) and ST22 (10/74, 13.51%, comprising 8 MRSA and 2 MSSA) as the predominant strains in Ningxia, which aligns with findings from prior research conducted in other regions of China [29,30]. Furthermore, it is suggested that ST59 MRSA may transition from community settings to hospitals [31,32]. warranting increased vigilance regarding its dissemination in healthcare facilities. Additionally, the predominant SCC*mec* type identified was type IVa (77.61%), a result that is consistent with classifications observed in the eastern region of Heilongjiang Province and Hainan [10,33]. The prevalence of the dominant clonal strain ST59-IVa MRSA in Ningxia (46.27%) corresponds with trends reported in national studies across China in recent years [9]. This report indicates that the previously dominant ST239-III strain has been supplanted by a gradual rise in the detection rates of ST59-IV and ST5-II. Notably, No ST239 isolates, which are associated with hospital-acquired infections in China, were detected in this study., while the emergence of ST59-IVa as the dominant strain corroborates findings from earlier studies.

### 4.2 MRSA resistance mechanisms and virulence genes

The resistance mechanism of MRSA is mainly obtained by integrating the *mecA* gene into specific sites on the chromosome [34]. Our study found that 91.04% of MRSA strains carried *mecA*, while none of the seven MSSA strains did. Notably, the five ST22-IVa strains exhibited a distinct resistance profile, demonstrating resistance to β-lactams and macrolides despite the absence of *mecA* and *erm* genes. This genotype–phenotype discordance aligns with the typical features of borderline oxacillin-resistant *Staphylococcus aureus* (BORSA). BORSA phenotypes are predominantly attributed to either hyperproduction of the β-lactamase *blaZ* (encoded by the *blaZ* gene) or specific point mutations in native penicillin-binding proteins (e.g., PBP4) [35,36]. In the present study, all five ST22-IVa isolates harboured the *blaZ* gene (Table 2, Fig 4), leading us to speculate that *blaZ* hyperproduction may act as the primary driver of drug resistance in these isolates. Notably, the co-occurrence of a hypervirulent genotype (*PVL ⁺ /tsst-1*⁺) and the BORSA phenotype in ST22-IVa strains represents a critical clinical and public health concern, which merits further in-depth exploration.

In addition to the atypical β‑lactam resistance observed in ST22‑IVa, the aminoglycoside resistance genes *aph(3’)-III* and *ant(6)-Ia* were detected in multiple ST59 and ST3355 isolates (Table 2), yet all remained phenotypically susceptible to aminoglycosides. Such genotype–phenotype inconsistency is common in *Staphylococcus aureus*. A study of 1,470 *S. aureus* isolates reported that 10.3% carried silent resistance genes, defined as silencing of antibiotic resistance by mutation (SARM), mostly caused by frameshift mutations in poly(A) tracts [37]. Similar frameshift-mediated silencing has been reported for *mupA*, leading to premature truncation and loss of resistance phenotype [38]. Therefore, genotype–phenotype discordance may result from gene silencing, low transcription, or nonfunctional alleles. Further functional studies are needed to confirm the exact mechanisms underlying these observations.

MRSA strains exhibit a variety of virulence factors that are integral to their ability to colonize, invade, and cause systemic infections [39–41].In the present study, the PVL carriage rate of strain ST59 was only 9.68%, while that of MRSA-ST22 was 87.5% (7/8). The difference in PVL prevalence between the two strains of the same CA-MRSA may be a result of the evolutionary variation of MRSA due to various factors. In a study of MRSA strains, the detection rate of the *tsst-1* gene was found to be 13.5% [42]. In this study, the detection rate of *tsst-1* was (6/67, 8.96%), which is lower than other studies. Furthermore, the coexistence of the PVL and *tsst-1* genes was observed in five ST22-IVa MRSA strains, indicating a strong virulence potential [43], consistent with the virulence profile of ST22-IVa strains identified in a Chinese hospital by Zhao Huilin [44] et al. who reported that such strains accounted for up to 18% of invasive infections. Seven enterotoxins were detected in the ST22 strain, which carried more enterotoxin species compared to ST59 and ST3355. Consequently, there is an imperative to monitor the prevalence and dissemination of ST22 to mitigate the potential adverse effects of highly virulent strains on public health. This underscores the necessity for enhanced surveillance of such virulent strains in future research endeavors.

### 4.3 Genetic evolution and diversity

The present study indicates a close genetic relationship between ST59, ST3355, and ST4513, with a core genome divergence of merely 3–5 single nucleotide polymorphisms (SNPs) between ST3355 and ST59 strains. Both strains exhibit SCC*mec* types IVa, implying that ST3355 may represent a subclone derived from ST59 due to a mutation (allele 283) in the *gmk* locus. This observation suggests that this clone possesses a significant potential for rapid adaptive evolution within the hospital environment of Ningxia. These findings align with the mutation trends of ST59 as reported by Wang et al [9]. In contrast, the ST5 lineage has given rise to nine distinct ST types through multilocus mutations, demonstrating considerable genomic plasticity; but did not become a locally dominant clone. This phenomenon contrasts with the report of ST5-II type dominance in Hainan [10], suggesting that regional medical resources may significantly influence clonal selection in MRSA. For example, the widespread use of β-lactams in primary care in Ningxia may have accelerated the localization dominance of ST59-Iva [7]. In addition, differences in infection control policies, population density, and inter‑hospital patient transfer may also contribute to clonal replacement patterns. Future studies integrating antimicrobial consumption data with patient movement trajectories would help elucidate these contributing factors.

### 4.4 Study limitations and future directions

This study encompasses only 74 strains collected during the first half of 2024, representing a relatively small sample size and a restricted temporal scope. Consequently, this limitation may hinder the adequate detection of certain low-prevalence clonotypes. Our phylogenetic tree lacked recombination filtering and temporal calibration, so evolutionary relationships should be interpreted cautiously. Furthermore, short-term data may not effectively capture seasonal variations or long-term evolutionary trends associated with MRSA. Future research should incorporate multi-center and inter-annual sampling, coupled with temporal and spatial dynamic analyses, to provide a more comprehensive understanding of the epidemiological characteristics of MRSA in Ningxia.

## 5 Conclusion

This research indicated that the predominant strain of MRSA in the hospital environment of Ningxia is the ST59-IVa type, which aligns with the prevailing domestic epidemiological patterns regarding multi-drug resistance and virulence gene profiles. However, the heightened virulence traits and atypical resistance mechanisms associated with the five ST22-IVa type strains warrant attention. It is advisable for healthcare facilities in the Ningxia region to enhance molecular surveillance of hypervirulent ST22-IVa strains and to integrate them into standard infection control protocols. Additionally, it is recommended to optimize the usage guidelines for β-lactams to mitigate the risk of resistance transmission.

## Supporting information

S1 File **Table S1.** Inclusion and exclusion criteria for patient and isolate selection. **Table S2**. Clinical and epidemiological characteristics of each S. aureus isolate with CA/HA classification. **Figure S1**. Phylogenetic tree of 74 S. aureus isolates with scale bar.
(DOCX)

## Abbreviations

MRSA: Methicillin-resistant *Staphylococcus aureus*
HA-MRSA: hospital-acquired MRSA
CA-MRSA: community-acquired MRSA
PVL: leukocidin Panton-Valentine
tsst-1: toxic shock syndrome toxin-1
MLST: Multi locus sequence typing
SCCmec: Staphylococcal Cassette Chromosome mec
