# Correction: Methicillin-resistant *Staphylococcus aureus* (MRSA) infection in hospitalized patients is dominated by community-acquired strains: genomic epidemiological evidence

**DOI:** 10.1371/journal.pone.0357884

**Published:** 2026-09-03

**Authors:** Jingxia Dang, Yuhui Geng, Ting Pan, Ping Zhang, Mingbo Chen, Dongfeng Pan, Peifeng Liang

The affiliations are listed out of order. Please view the correct order of the affiliations here:

Jingxia Dang^1,3^, Yuhui Geng⁴, Ting Pan^1,3^, Ping Zhang^1,3^, Mingbo Chen^1,3^, Dongfeng Pan^5^, Peifeng Liang^2^

**1** School of Public Health, Ningxia Medical University, Yinchuan, China, **2** Department of Medical Affairs, People’s Hospital of Ningxia Hui Autonomous Region, Ningxia Medical University, Yinchuan, China, **3** Ningxia Key Laboratory of Environmental Factors and Chronic Disease Control, Yinchuan, China, **4** Department of Infection Control, General Hospital of Ningxia Medical University, Yinchuan, China, **5** Department of Emergency Medicine, People’s Hospital of Ningxia Hui Autonomous Region, Ningxia Medical University, Yinchuan, China.

## References

[1] Dang J, Geng Y, Pan T, Zhang P, Chen M, Pan D, et al. Methicillin-resistant *Staphylococcus aureus* (MRSA) infection in hospitalized patients is dominated by community-acquired strains: genomic epidemiological evidence. PLoS One. 2026;21(7):e0354017. doi: 10.1371/journal.pone.0354017 42531211 PMC13422852

