# Supplementary material for: Methicillin-resistant Staphylococcus aureus (MRSA) infection in hospitalized patients is dominated by community-acquired strains: genomic epidemiological evidence
Source: PLoS One. 2026 Jul 30;21(7):e0354017. doi: 10.1371/journal.pone.0354017 (PMC13422852; doi:10.1371/journal.pone.0354017)

**Supplementary Material**

**For: “****Methicillin-resistant *Staphylococcus aureus* (MRSA) infection in hospitalized patients is dominated by community-acquired strains: genomic epidemiological evidence”**

**Table S1.** Inclusion and exclusion criteria for patient and isolate selection

**Table S2.** Clinical and epidemiological characteristics of each S. aureus isolate with CA/HA classification

**Figure S1.** Phylogenetic tree of 74 *S. aureus* isolates with scale bar

**Supplementary Table S1.** **Inclusion and exclusion criteria for patient and isolate selection**

| Category | Criteria |
| --- | --- |
| Inclusion criteria | 1. Patients with clinical signs of active infection (fever, elevated inflammatory markers, localizing signs)  2. Positive culture for *S. aureus* from an appropriate clinical specimen (sputum, pus, wound secretions, blood, or other sterile site fluids).  3. Isolate obtained before or within 48 hours of initiation of antibiotic therapy (when applicable). |
| Exclusion criteria | 1. Duplicate isolates from the same patient within a 30-day period.  2. Isolates considered colonization (positive culture without any corresponding clinical signs of infection).  3. Patients who had received broad-spectrum antibiotic therapy for more than 48 hours prior to specimen collection (to minimize false-negative cultures). |

**Supplementary Table S2.** **Clinical and epidemiological characteristics of each S. aureus isolate with CA/HA classification.**

| sample | gender | | age | | Length  of stay(days) | | Source of specimen | | Specimen Collection Date | | Admission Date | | MRSA Type | |  |
| --- | --- | --- | --- | --- | --- | --- | --- | --- | --- | --- | --- | --- | --- | --- | --- |
| N807 | | female | | 57 | | 7 | | pus | | 2023/12/04 | | 2023/12/4 | | CA-MRSA | |
| N810 | | male | | 51 | | 63 | | exudate | | 2023/12/11 | | 2023/12/7 | | HA-MRSA | |
| N811 | | male | | 55 | | 7 | | pus | | 2023/12/12 | | 2023/12/12 | | CA-MRSA | |
| N814 | | female | | 53 | | 40 | | others | | 2023/12/22 | | 2023/12/21 | | CA-MRSA | |
| N816 | | male | | 28 | | 7 | | sputum | | 2024/01/01 | | 2024/1/1 | | CA-MRSA | |
| N821 | | male | | 53 | | 28 | | sputum | | 2024/01/06 | | 2023/12/15 | | HA-MRSA | |
| N823 | | male | | 60 | | 9 | | blood | | 2024/02/01 | | 2024/1/10 | | HA-MRSA | |
| N827 | | male | | 17 | | 5 | | sputum | | 2024/01/12 | | 2024/1/11 | | CA-MRSA | |
| N829 | | female | | 70 | | 21 | | sputum | | 2024/01/15 | | 2024/1/12 | | HA-MRSA | |
| N830 | | male | | 45 | | 19 | | others | | 2024/01/16 | | 2024/1/11 | | HA-MRSA | |
| N831 | | male | | 2 | | 6 | | others | | 2024/01/16 | | 2024/1/16 | | CA-MRSA | |
| N833 | | female | | 70 | | 14 | | exudate | | 2024/01/20 | | 2024/1/19 | | CA-MRSA | |
| N834 | | male | | 16 | | 6 | | exudate | | 2024/01/19 | | 2024/1/19 | | CA-MRSA | |
| N837 | | male | | 34 | | 2 | | pus | | 2024/01/23 | | 2024/1/22 | | CA-MRSA | |
| N838 | | male | | 96 | | 7 | | sputum | | 2024/01/25 | | 2024/1/19 | | HA-MRSA | |
| N840 | | male | | 62 | | 37 | | blood | | 2024/01/26 | | 2024/1/26 | | CA-MRSA | |
| N842 | | male | | 67 | | 28 | | pus | | 2024/01/29 | | 2024/1/29 | | CA-MRSA | |
| N843 | | female | | 61 | | 12 | | sputum | | 2024/01/29 | | 2024/1/27 | | CA-MRSA | |
| N845 | | male | | 74 | | 15 | | exudate | | 2024/02/02 | | 2024/1/29 | | HA-MRSA | |
| N850 | | male | | 47 | | 56 | | others | | 2024/02/13 | | 2024/2/12 | | CA-MRSA | |
| N856 | | female | | 86 | | 15 | | sputum | | 2024/02/22 | | 2024/2/21 | | CA-MRSA | |
| N857 | | male | | 44 | | 15 | | sputum | | 2024/02/17 | | 2024/2/13 | | HA-MRSA | |
| N860 | | male | | 84 | | 12 | | sputum | | 2024/05/03 | | 2024/5/2 | | CA-MRSA | |
| N862 | | male | | 32 | | 22 | | pus | | 2024/03/07 | | 2024/3/6 | | CA-MRSA | |
| N868 | | male | | 37 | | 6 | | sputum | | 2024/03/17 | | 2024/3/17 | | CA-MRSA | |
| N870 | | male | | 72 | | 16 | | sputum | | 2024/03/24 | | 2024/3/23 | | CA-MRSA | |
| N871 | | male | | 66 | | 62 | | sputum | | 2024/03/25 | | 2024/2/14 | | HA-MRSA | |
| N877 | | male | | 0 | | 13 | | sputum | | 2024/03/29 | | 2024/3/23 | | HA-MRSA | |
| N880 | | male | | 51 | | 5 | | others | | 2024/04/01 | | 2024/3/30 | | CA-MRSA | |
| N891 | | male | | 52 | | 20 | | pus | | 2024/04/10 | | 2024/4/10 | | CA-MRSA | |
| N898 | | female | | 34 | | 12 | | others | | 2024/04/25 | | 2024/4/24 | | CA-MRSA | |
| N899 | | male | | 37 | | 25 | | others | | 2024/04/25 | | 2024/4/21 | | HA-MRSA | |
| N902 | | male | | 36 | | 7 | | sputum | | 2024/04/30 | | 2024/4/29 | | CA-MRSA | |
| N911 | | female | | 31 | | 9 | | others | | 2024/05/13 | | 2024/5/9 | | HA-MRSA | |
| N912 | | male | | 15 | | 11 | | sputum | | 2024/05/12 | | 2024/5/11 | | CA-MRSA | |
| N915 | | male | | 54 | | 60 | | exudate | | 2024/05/17 | | 2024/5/17 | | CA-MRSA | |
| N923 | | male | | 30 | | 30 | | others | | 2024/05/20 | | 2024/5/13 | | HA-MRSA | |
| N925 | | female | | 1 | | 10 | | exudate | | 2024/05/26 | | 2024/5/26 | | CA-MRSA | |
| N928 | | male | | 59 | | 12 | | exudate | | 2024/05/29 | | 2024/5/29 | | CA-MRSA | |
| N931 | | male | | 29 | | 7 | | exudate | | 2024/05/30 | | 2024/5/30 | | CA-MRSA | |
| N934 | | male | | 0 | | 92 | | others | | 2024/06/01 | | 2024/5/6 | | HA-MRSA | |
| N939 | | male | | 29 | | 7 | | sputum | | 2024/06/02 | | 2024/5/27 | | HA-MRSA | |
| N942 | | male | | 52 | | 10 | | others | | 2024/06/08 | | 2024/6/6 | | CA-MRSA | |
| N944 | | female | | 79 | | 26 | | sputum | | 2024/06/05 | | 2024/5/16 | | HA-MRSA | |
| N945 | | female | | 69 | | 66 | | sputum | | 2024/06/10 | | 2024/6/5 | | HA-MRSA | |
| N946 | | male | | 42 | | 10 | | exudate | | 2024/06/11 | | 2023/7/4 | | HA-MRSA | |
| N950 | | male | | 64 | | 31 | | sputum | | 2024/06/12 | | 2024/6/4 | | HA-MRSA | |
| N953 | | male | | 76 | | 11 | | blood | | 2024/06/17 | | 2024/6/16 | | CA-MRSA | |
| N954 | | male | | 47 | | 48 | | sputum | | 2024/06/20 | | 2024/6/16 | | HA-MRSA | |
| N957 | | female | | 75 | | 8 | | sputum | | 2024/06/20 | | 2024/6/16 | | HA-MRSA | |
| N960 | | male | | 70 | | 16 | | sputum | | 2024/06/24 | | 2024/6/15 | | HA-MRSA | |
| N961 | | male | | 42 | | 7 | | pus | | 2024/06/24 | | 2024/6/24 | | CA-MRSA | |
| N963 | | female | | 80 | | 8 | | exudate | | 2024/06/24 | | 2024/6/24 | | CA-MRSA | |
| N969 | | male | | 68 | | 15 | | sputum | | 2024/06/30 | | 2024/6/29 | | CA-MRSA | |
| N973 | | male | | 65 | | 18 | | sputum | | 2024/07/03 | | 2024/6/28 | | HA-MRSA | |
| N977 | | male | | 59 | | 36 | | blood | | 2024/07/08 | | 2024/6/11 | | HA-MRSA | |
| N980 | | female | | 62 | | 23 | | blood | | 2024/07/09 | | 2024/6/24 | | HA-MRSA | |
| N982 | | male | | 0 | | 60 | | sputum | | 2024/07/10 | | 2024/7/8 | | CA-MRSA | |
| N983 | | male | | 44 | | 18 | | blood | | 2024/07/10 | | 2024/7/10 | | CA-MRSA | |
| N986 | | female | | 54 | | 17 | | blood | | 2024/07/10 | | 2024/7/2 | | HA-MRSA | |
| N987 | | female | | 70 | | 7 | | sputum | | 2024/07/10 | | 2024/7/10 | | CA-MRSA | |
| N991 | | male | | 31 | | 10 | | exudate | | 2024/07/10 | | 2024/7/10 | | CA-MRSA | |
| N992 | | male | | 35 | | 13 | | others | | 2024/07/10 | | 2024/7/16 | | CA-MRSA | |
| N998 | | female | | 41 | | 17 | | blood | | 2024/07/23 | | 2024/7/23 | | CA-MRSA | |
| N1000 | | male | | 60 | | 7 | | others | | 2024/07/24 | | 2024/7/22 | | CA-MRSA | |
| N1002 | | female | | 93 | | 8 | | pus | | 2024/07/24 | | 2024/7/26 | | CA-MRSA | |
| N1004 | | male | | 0 | | 8 | | exudate | | 2024/08/03 | | 2024/7/31 | | HA-MRSA | |
| N828 | | female | | 16 | | 8 | | exudate | | 2024/01/22 | | 2024/1/22 | | MSSA | |
| N861 | | male | | 75 | | 43 | | others | | 2024/03/06 | | 2024/3/4 | | MSSA | |
| N873 | | female | | 27 | | 3 | | others | | 2024/03/25 | | 2024/3/25 | | MSSA | |
| N893 | | male | | 34 | | 15 | | exudate | | 2024/04/14 | | 2024/4/13 | | MSSA | |
| N900 | | male | | 72 | | 14 | | exudate | | 2024/04/27 | | 2024/4/26 | | MSSA | |
| N905 | | male | | 58 | | 30 | | exudate | | 2024/05/07 | | 2024/5/7 | | MSSA | |
| N996 | | female | | 65 | | 11 | | sputum | | 2024/07/21 | | 2024/7/21 | | MSSA | |

**Supplementary Figure S1.** **Phylogenetic tree of 74 *S. aureus* isolates with scale bar**


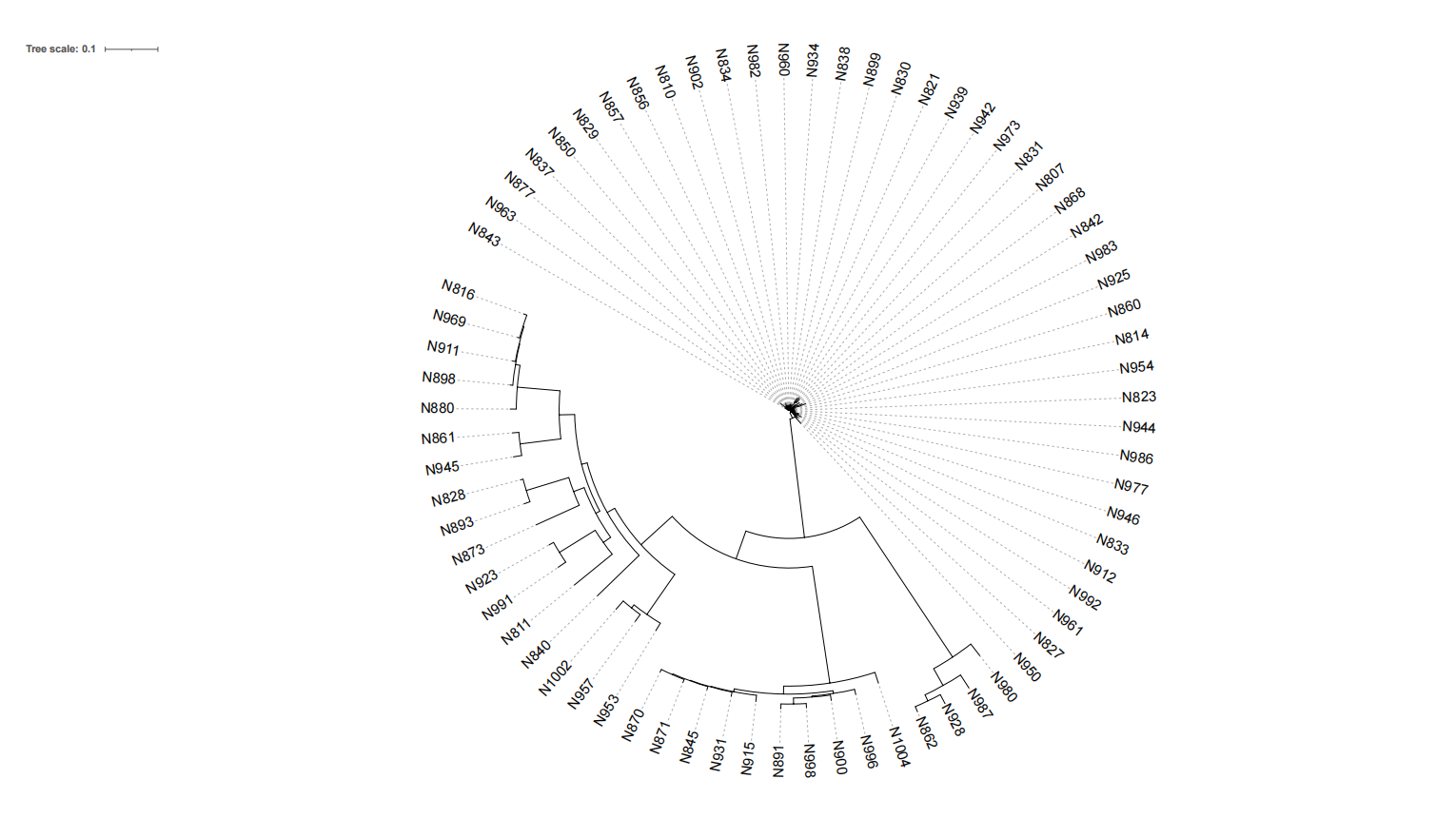

Supplement: S1 File — Table S1. Inclusion and exclusion criteria for patient and isolate selection. Table S2. Clinical and epidemiological characteristics of each S. aureus isolate with CA/HA classification. Figure S1. Phylogenetic tree of 74 S. aureus isolates with scale bar. (DOCX) [file pone.0354017.s001.docx]
